# The “hidden” epidemic: a snapshot of Moroccan intravenous drug users

**DOI:** 10.1186/1743-422X-11-43

**Published:** 2014-03-06

**Authors:** Roxana-Delia Trimbitas, Fatima Zahra Serghini, Fatiha Lazaar, Warda Baha, Abderrahim Foullous, Mohammed Essalhi, Abdelouahed El Malki, Abdelkrim Meziane Bellefquih, Abdelouaheb Bennani

**Affiliations:** 1 Pasteur Institute of Morocco, Place Louis Pasteur, 20360 Casablanca, Morocco; 2 Hasnouna Center of Addictology, 15 Moulay Driss St., Tangier, Morocco; 3 Delegation of Public Health (Tangier/Tetouan region), Ministry of Health, Tetouan Public Hospital, Av. Abdelkhalek Torres, Tetouan, Morocco; 4 Department of Medical Biology, Molecular Biology Laboratory, Pasteur Institute of Morocco, Place Louis Pasteur, Casablanca, Morocco

**Keywords:** Hepatitis C, IDUs, Prevalence, Genotype, Risk factors, Public health, Morocco

## Abstract

**Background:**

Hepatitis C virus is a persistent epidemiological problem, with an estimated 170 million individuals infected worldwide, and the leading cause of asymptomatic chronic infection, liver cirrhosis and hepatocellular carcinoma. Injection drug users (IDUs) have the highest seroprevalence as compared to chronic hemodialysis and transfusion patients, and this cohort remains the most under-studied high-risk group in North Africa to date. This study first sought to characterize the demographic, epidemiological, and genotypic profile of a total sample size of 211 chronically-infected IDUs living in the Tangier region of Northern Morocco, and secondly to contrast this to other chronically-infected patients, in order to uncover possible discrepancies.

**Results:**

The general ‘profile’ of local IDUs marks a stark contrast to chronically-infected HCV Moroccan patients, other African countries, and neighboring European countries. The majority of Moroccan drug users were found to be middle-aged and celibate. A relatively high seroprevalence was found among drug users (60%), and this increased with age. The majority of drug users shared their needles and this hold implications for transmission, as seropositive status was significantly different between those users that shared vs. those that did not share their needles. In addition, IDUs exhibited genotypes 1a and 3a predominantly, as compared to the predominant 1b and 2a/2c genotypes found in chronically HCV-infected patients. The IDU genotypic profile closely matches the one in other European countries (Portugal, Spain, France, and Italy), which are invariably speculated as the potential source of currently-circulating genotypes in Moroccan IDUs.

**Conclusion:**

These findings have implications for disease prevention, transmission and treatment, as this distinct IDU subgroup cannot be collectively pooled along with other HCV-positive high-risk groups. Local government, practitioners, and health institutions should take this into account when treating, prescribing antiviral therapy, and designing preventative public health campaigns.

## Introduction

Since its discovery in the 1980s, Hepatitis C Virus (HCV) has caused significant mortality and morbidity with an estimated 170 million people infected worldwide (Aitken et al.) [1]. Even though this blood-borne virus has received less spotlight than other infectious virus counterparts such as HIV-1, it has infected nearly four times as many people and is expected to overtake the number of death resulting from liver-related damage and hepatocellular carcinoma (HCC) as by AIDS (Cohen) [2]. Only 25% of infected individuals will clear the virus within the first 6 months, and thus, this low clearance translates into most infected individuals becoming chronic carriers. The acute infection phase is mostly asymptomatic or with mild non-specific symptoms such as lethargy and myalgia, result in that many cases remain undetected for years (Grebley et al.) [3]. Modern day combination triple therapy used to treat HCV infection is composed of pegylated interferon-alpha, ribavirin and direct acting antivirals, the cost of which averages around US$1,100 per week and comes with its fair share of side effects (Mole) [4]. Furthermore, the virus’ high mutation rate, rapid evolution and high genetic diversity complicate the task of effective vaccine development and collectively has resulted in no marketed vaccine to date (Liang) [5].

The Arab world is a very large and vast region, encompassing the Arabian Peninsula, Sham region, Arabian Nile region, and the North African region, is no exception to this epidemic and is estimated to harbour 25 million infected individuals and an average prevalence rate of 3.5%. Illegal drug use along with sexual activity outside of wedlock fall into the boat of taboo topics resulting in that investigators having shunned this research area until recent years (Daw & Dau) [6]. Further complicating the matter is the low level of government funding for epidemiological research activities, ultimately resulting in an unconfirmed true prevalence rate for most countries in this region.

Of all the countries in the world, Egypt has the highest prevalence of HCV estimated at 14.7% (Mohamoud et al.) [7], which has been ravaging the country for decades and is thought to be mainly spreading through iatrogenic transmission (Miller) [8]. Closer to home, the overall prevalence of HCV in the general Moroccan population is lower and classified as ‘intermediate’, currently estimated at 1.58% and 1.93% (Baha et al.; Benouda et al.) [9,10]. We note a discrepancy with the reported prevalence rate from the previous decade, which was estimated to be 7.7% in patients hospitalized for various pathologies (Cacoub et al.) [11]. We note that there might be a bias and that this discrepancy in prevalence might be due to the fact that the group of patients included the Cacoub study was a selective and a more ill group and did not represent the general Moroccan population.

The HCV situation is more contained in Morocco due to the routine blood screening strategies implemented 20 years ago (Baha et al.) [9]. Presently, the major high-risk groups in the country are hemodialysis and hemophiliac patients, and injecting drug users. It is now globally accepted that the IDU cohort has the highest prevalence among all the high-risk groups (ranging from 60-90%), serves as a reservoir for the blood-borne virus, and accounts for the overwhelming majority of new transmissions (Backmund et al.) [12]. This issue is further aggravated by the fact that the majority of IDUs are unaware of their seropositive status and do not routinely seek treatment (Miller) [13]. Data on IDU seroprevalence is only available in 7 countries in the Middle East (Cyprus, Egypt, Iran, Israel, Lebanon, Saudi Arabia, and Syria), yet the genotype distributions and complete profile for each country are incomplete (Ramia et al.) [14].

No published data on the Moroccan IDU cohort exists to date, despite the fact that there is extensive drug trafficking on the Northern border between Spain. The sparse and fragmentary nature of published data on the Maghreb region (Morocco, Mauritania, Algeria, and Tunisia) has resulted in a lack of thorough understanding and hinders the planning of strategic long-term prevention strategies. The primary aim of this study was to characterize the somewhat ‘hidden’ IDU cohort of Morocco in terms of their demographics, seroprevalence and genotype profile. By targeting this high-risk group, tailored risk-management and long-term preventative strategies can be developed and implemented with the dual aim of lowering the HCV national prevalence rate and ultimately lessening the financial and socio-economic burden of future generations.

## Results

Our study used a total sample size of 211 IDU patients, and data collection on the various variables under study was done over a 2-year period. General inclusion criteria into the study cohort depended on the availability of a minimum of one of the following relevant data variables: age/year of birth; gender; marital status; needle-sharing habits (Yes or No); route of drug administration; HCV seropositivity status (positive or negative); HCV genotype; HCV viral load. Every effort was made to compile a complete data set including data on all variables for each patients, however, the difficulty involved in doing this resulted in the ‘conditional inclusion’ of patients in a statistical test based on the availability of the needed respective variables (pairwise deletion). The data is assumed to be missing completely at random, and subjects with missing observations for certain measures were excluded from some tests, while included in others. We sought to maximize the use of all available data and in this way make use of a ‘rotating’ sub-sample taken selectively from our total sample size. Analysis results are presented along with the number of patients included in each statistical test in question, and all p values inferior to 0.05 were considered significant.

### Demographic variables

The average age of IDUs was found to be 38.5 ± 8.7 (based on available year of birth data, *n* = 154), the median was 40 years old, with an age range of 39 years (19 to 58 years old). Gender data was available for 167 subjects, with the sex ratio of males to females was 148/19 = 7.78. Civil status (*n* = 166 subjects): the majority of subjects were single (119/166, 71.6%), while the remaining were married (24/166, 14.5%), divorced (16/166, 9.6%), separated (5/166, 3%), and widowed (2/166, 1.2%) respectively.

For HCV chronically infected subjects: the mean age of patients was 59.8 ± 13.8 years (*n* = 151), and the median 60 years old. The youngest patient was 18 years old while the oldest was 86 years old, yielding an age range of 68 years. The sex ratio of males to females was 51/100 = 0.51. No civil status data was available for chronic patients. Average age of women (*n* = 100) was 59.7 ± 13.4 years and the average age of men (*n* = 51) was 60.2 ± 14.7 years respectively.

### Epidemiological variables

**Figure 1:**
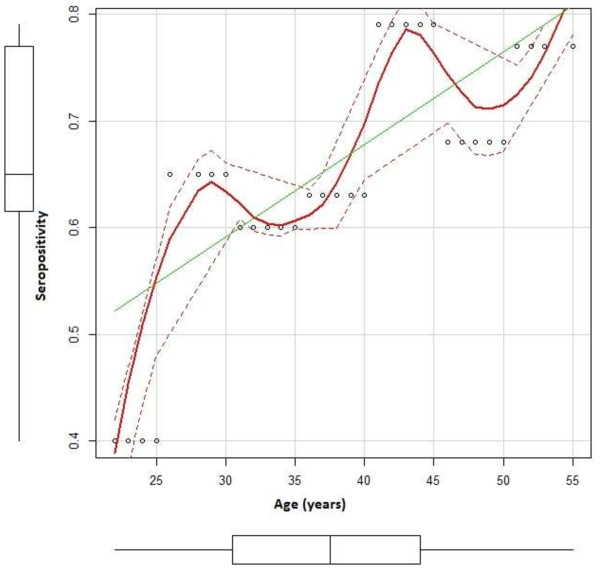
**Title: positive correlation between age and average seropositivity.** Legend: Subjects were binned into 7 age groups (21-25; 26-30; 31-35; 36-40; 41-45; 46-50; 51-55) and average seropositivity was calculated per age group. Pearson’s Product Moment reveals a moderately positive correlation between age and seropositivity (r = 0.726 95% CI [0.633, 0.798]). Seropositivity (proportion) is specified on the Y-axis, while age groups are specified on the X-axis. Individual data points are shown in circles, while average seropositivity per age group is shown by the red line. Best-fit line upon which the correlation is based is indicated in green.

The prevalence of HCV in our cohort was calculated based on available viral load data obtained via RT-PCR, where the patient was classified as positive if harbouring a viral load higher than the limit of detection of 15 IU/mL. The proportion of IDU patients seropositive for HCV was 89/148 = 0.60 (*n* =148). Age and seropositivity (*n* = 132 IDU subjects) were moderately correlated (R = 0.726, 95% CI [0.633, 0.798]), as depicted in Figure 1. The average viral load was calculated to be 2.66 × 10^6^ ± 4.53 × 10^6^ IU/mL for IDUs (*n =* 49) and 2.79 × 10^6^ ± 6.10 × 10^6^ IU/mL for chronically infected patients (*n =* 151). Independent samples t-test for the comparison of average viral load between IDUs and chronic patients, using the unequal variance approach (F _[1.813]_, p = 0.009) was non-significant (t = 0.159, *df* = 109.46, p = 0.4369). *Risk factors*: Drug administration route was assessed and revealed that a minority of drug addicts (8/101, 0.08%) smoke the heroine as opposed to shooting it. Needle-sharing habits showed that the overwhelming majority of IDUs shared their needles (86/101 = 85.1%, *n =* 101). Chi-squared analysis for needle sharing habits vs. seropositive status was significant (χ^2^ = 43.22, p = 4.87e^-11^).

### Genotype profiles

**Figure 2:**
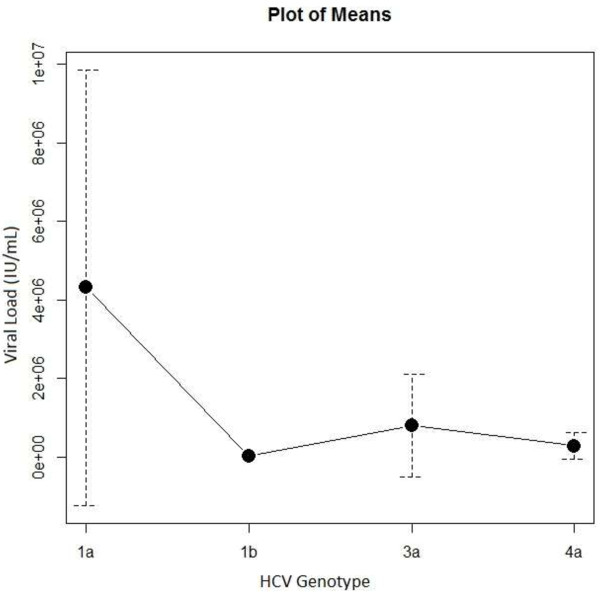
**Title: average viral load (IU/mL) per genotype (IDU).** Legend: Plot of means of IDU genotype distribution depicting average viremia (IU/mL) and standard deviations. No standard deviation given for genotype 1b as only one subject was available for assessment. Genotype: 1a (*n* = 27, x̅ = 4.3 x 10^6^ ± 5.5 x 10^6^ IU/mL); 1b (n = 1, x̅ = 1.7 x 10^4^ IU/mL); 3a (n = 16, x̅ = 8.04 x 10^5^ ± 1.3 x 10^6^ IU/mL); 4a (n = 5, x̅ = 2.8 x 10^6^ ± 3.4 x 10^5^ IU/mL). Genotype 1a shows the highest average viremia and standard deviation as compared to all other genotypes, however the highest dispersion might be due to a large number of subjects in this catergory.

IDUs and chronically infected patients’ genotypic distribution of HCV differs substantially. The majority of IDUs (*n* = 74) carry genotype 1a (60.8%) and 3a (25.6%), with a smaller number carrying genotypes 1b (4.05%) and 4 (9.4%) respectively, as seen in Table 1. The predominant genotypes exhibited by our IDUs matched closely with several countries hugging the Mediterranean basin (Table 2). Patients were grouped into 7 age intervals in order to examine the relative genotype frequencies among age groups, the results of which can be seen in Table 3. Genotype 1a seems to be present in all ages, while genotype 3a seems to be present in younger IDUs and genotype 1b in older ones respectively. Genotype 4 remains infrequent, which closely approximates the general Moroccan population. Chi-squared analysis of genotype profile compared between IDUs and chronically infected patients was significant (χ^2^ = 151.1, *df =* 5, p = 2.2e^-16^). ANOVA analysis of viral load (IU/mL) as a response variable to genotype was significant (F = 2.98, p = 0.0412, *n* = 49), indicating that IDUs with genotype 1a had the highest viral load as compared to all other genotypes (4.3 × 10^6^ ± 5.5 × 10^6^), as displayed in Figure 2.

**Table 1:** HCV Genotype distribution among intravenous drug users (IDUs) and chronically-infected patients

**Genotype**	**Frequency count (IDU)**	**%**	**Frequency count (Chronic patients)**	**%**
Genotype 1a	45	60.8	6	5.2
Genotype 1b	3	4.05	45	39.1
Genotype 1nc	0	0	2	1.7
Genotype 2	0	0	44	38.2
Genotype 2nc	0	0	3	2.6
Genotype 3	19	25.6	9	7.8
Genotype 4	7	9.4	6	5.2
Genotype 5	0	0	0	0
Genotype 6	0	0	0	0
Total	74	100	115	100

**Table 2:** Reported IDU genotype profile per country in North Africa, Europe, and the Mediterranean basin

**Country**	**Genotype (%)**	**Reference**
Lebanon	3a (57%), 1a (21%), 4 (18%)	Mahfoud et al. [15]
Greece	3a (59%), 1 (23%)	Raptopoulou et al. [16]
Croatia	1b (61%), 3a (26%)	Vince et al. [17]
Serbia	1b (41.4%), 3a (27.6%)	Stamenkovic et al. [18]
Italy	1a (30%), 3a (40%), 1b (11%)	Ciciarello et al. [19] ; Stroffolini et al. [20]
France	1b (27%), 3a (21%), 1a (18%)	Payan et al. [21]
Spain	1a (42.8%), 3a (20.6%)	Alonso Alonso et al. [22];
Portugal	1a (64.9%), 3a (71.6%)	Almeida Calado et al. [23]

Percentages of respective genotype are provided in brackets when available.

**Table 3:** Distribution of HCV genotypes across various age group

	**HCV genotype**			
**Age (n)**	**1a**	**1b**	**3a**	**4a**
16-20 (4)	1 (0.25)	0	3 (0.75)	0
21-25 (7)	5 (0.71)	0	1 (0.14)	1 (0.14)
26-30 (10)	6 (0.60)	0	3 (0.30)	1 (0.10)
31-35 (13)	7 (0.53)	1 (0.07)	1 (0.07)	4 (0.31)
36-40 (12)	7 (0.58)	2 (0.16)	3 (0.25)	0
41-45 (11)	8 (0.73)	3 (0.27)	0	0
46-50 (2)	2 (1.00)	0	0	0

IDU subjects (*n* = 59) were grouped into 7 age intervals, whereas number of subjects per group are provided in brackets. The frequency counts of genotypes observed is provided as well as the proportions in parentheses.

## Discussion

While several published Moroccan studies exist in the scientific literature on hemodialysis, haemophiliacs and chronic HCV-infected patients (Sekkak et al.; Benouda et al.; Benani et al.; Benjelloun et al.) [10,24-26], the IDU group in the Maghreb region has remained a largely under-studied cohort to date. Due to the paucity of published data, this study sought to characterize this elusive and high-risk group, as well as to elucidate key difference between it and other chronically HCV-infected patients in Morocco.

The average age of IDUs (*n* = 154) was found to be 38.5 ± 8.7 years and the median 40 years. The youngest and oldest subjects were 19 and 58 years old respectively, thereby our sample had an age range of 39 years. Only a small fraction of subjects were reportedly in a conjugal relationship (married, 14.5%), while the overwhelming majority (85.5%) were either single, divorced, widowed or separated. A large part of the subjects were male, with a male/female sex ratio of 7.78 (*n* = 167). This gender asymmetry may be explained in two ways: either that the majority of the injecting drug users in Morocco are indeed male and this is a predominantly male-associated drug habit, or that females hesitated to report their addiction because of social and cultural reasons.

In contrast, the chronically infected patients used as a comparator population had an average age of 59.8 ± 13.8 years (*n* = 151), with a median of 60 years old, indicating that his cohort was somewhat older than the IDUs. The age range was slightly larger as well (18 to 65 years, range of 47 years), as we speculate this is due the fact that there were older subjects present in the chronically infected group. Another demographic difference observed was the male to female sex ratio (51/100 = 0.51), as the majority of the chronic carriers were women. This skewedness did not bias the mean age of all patients, as women (*n* = 100) had an average age of 59.1 ± 13.4 years, and men (*n* = 51) an average of 60.12 ± 14.7 years respectively.

Slightly more than half of our IDU cohort was found to be anti-HCV positive (89/148, 60%), allowing us to classify them into and confirm the ‘highly seroprevalent’ stigma associated with IDUs worldwide. Previously reported studies in various parts of the world disclosed lower HCV-positive proportions in their local IDUs: Lebanon (50%) and Australia (45.8%) (Mahfoud et al.; Kynotch Aitken et al.) [1,15], while other studies reported higher HCV-positive proportions: San Francisco Bay Area, China, and Greece at 80.2%, 66.7% and 73% respectively (Gigi; Ucellini et al.; Bao & Liu) [27-29].

Age and seropositive status were shown to be positively correlated (Baha et al.; Benouda et al.; Mathei et al.) [9,10,30], in that older subjects (as a group) were more likely to be seropositive as compared to younger subjects (*n* =132). Due to our sufficient sub-sample size and wide range of ages, we were able to repeat the analysis by binning our IDU cohort into 7 age groups and calculating the average seropositivity for each group. Indeed, we report a moderately positive correlation between age and average seropositivity, confirming that the younger subjects were less likely to be anti-HCV positive (R =0.726) (Figure 1). As previously mentioned, Morocco implemented a national anti-HCV blood-screening practice in 1994 (Baha et al.) [9], and this is thought to have substantially contributed to the reduction of new HCV transmissions among younger generations. In addition, we also believe that the younger IDUs lower seropositivity can be explained by reduced duration of exposure to the risk associated with injecting drug use.

Already identified and well-studied risk factors associated with HCV transmission are incarceration, tattooing, needle-sharing, dental therapy, and a history of surgery (Baha et al.; Rice et al.) [9,31]. In terms of route of drug administration, only a small proportion (8/101 = 0.08%) of IDUs reported smoking as their habitual route of drug administration, as opposed to the more popular intravenous route. Five out of the eight users who smoked as their primary method for taking the drug were found to be anti-HCV positive, however, we note that the majority of the IDUs who smoked heroine were not subsequently screened for HCV-positive status. Further studies need to be done in with a sufficiently large sample size in order to establish route of drug administration as a *bone fida* risk factor, one which has implications for risk of infection.

The other major risk factor our study sought to address was the sharing of needles, with the majority of subjects reportedly doing so (86/101, 85.15%). This habit reflects the cultural aspect of the Moroccan and the larger Arab society, in the sense that these collectivist cultures are known for their sharing of food and goods as a symbol of bonding, brotherhood, and hospitality. One might also speculate that needle-sharing may be due to financial reasons during hard economic times, as the partage of one syringe between multiple users is less costly. The transmission of HCV is not only associated with contaminated syringes, but also with the drug preparation equipment, such cookers and filters, and the water in which the heroine is dissolved (Doerrbecker et al.) [32]. Not surprisingly, the act of needle-sharing was associated with a higher HCV-positive status among our cohort, and the χ^2^ test yielded significant results (χ^2^ = 43.22, p = 4.87e^-11^), leading us to conclude that the drug users under study who actively shared their needles were more likely to be HCV-positive as compared to those that did not.

We next sought to characterize possible serological and genotypic differences between IDUs and other chronically-infected patients. The average viral load (IU/mL) was not significantly different between IDUs and chronically-infected patients (t = 0.159, *df* = 109.46, p = 0.4369). Viral load is a factor routinely taken into account by practitioners when designing custom therapies for their patients. Pre-treatment viral load level has implications for treatment and is inversely related to treatment response, such that high viral load is often associated with a poor response to therapy (Amjad et al.) [33].

Perhaps the most striking difference between our IDU cohort and the chronically infected patients was their genotype profile (χ^2^ = 151.1, *df* = 5, p = 2.2e^-16^). The majority of IDUs harboured the genotypes 1a (60.8%), 3a (25.6%), while other chronically HCV mono-infected patients at the Pasteur Institute of Morocco in Casablanca primarily exhibited genotypes 1b and 2a/2c respectively (Table 1). A similar IDU profile has been documented in anti-HCV-positive subjects in other countries, some of which are in direct contact with Morocco and hugging the Mediterranean basin, such as Spain, Turkey, Croatia, and France (Payan et al.; Yildiz et al.; Vince et al.; Lopez-Labrador et al.) [17,21,34,35]. The Moroccan IDU profile does not match with neighbouring country Algeria or central Africa, which exhibit 1b, 2a/2c, and 4 as the predominant genotypes respectively (Rouabhia et al.; Xu et al.) [36,37], however we note that these cited studies were carried out in chronically infected and hemodialysis patients. One can speculate as to the multiple channels of heroin trafficking taking place across the Moroccan borders and the interactions and relationships that contribute to the distinct Moroccan IDU profile. Morocco shares a border with two neighbouring countries: Algeria on its Western front and Mauritania to the South. In the North, one simply crosses the Gibraltar strait to enter Spain and the European Union. Bordering on the East is the Atlantic Ocean, however direct access to the Spanish Canary Islands is possible via direct flights to Gran Canaria and Tenerife, two very popular touristic destinations. While the scientific literature lacks data on Mauritania, Algeria has an anti-HCV positive prevalence estimated at 0.02% (Lavancy; Rouabhia et al.) [36,38]. Interestingly, one Spanish study on both IDUs and chronically infected hemodialysis patients revealed that the majority of injecting drug users were genotype 1a (48.5%) (Garcia et al.) [39]. Finally, two drug user cohorts studied in Lisbon predominantly exhibited the genotypes 1a and 3a, resulting in this country harbouring a profile most similar to the local Moroccan drug users (Almeida Calando et al.) [23]. Collectively, these findings are clues and provide insight into the possible origin of local circulating genotypes among the drug users of Morocco. Available published data on HCV genotypes from IDUs living in countries close in geographic proximity to Morocco are displayed in Table 2.

Genotyping was performed on 59 out of the 154 IDUs who had age demographic data available, and patients were grouped into 7 age intervals (Table 3). At first glance, it seems that genotype 1a is found in all age groups, while genotypes 3a and 1b were present mostly in younger or older IDUs respectively. These differing proportions for genotypes 3a and 1b could be explained by a gradual shift in genotype profile over time and with older age groups having been exposed to different ‘sources’ of HCV as compared to younger generations. Genotype 1b has been found in the general population and among high-risk groups such as hemodialysis and blood transfusion patients (Benani et al.) [25], effectively classifying this genotype as ‘transfusion-related’. Since implementation of the national blood screening program in 1994 one would expect that younger subjects would have had a reduced risk of contracting this genotype, effectively explaining its absence in patients aged less than 30 years old. The low presence of genotype 4 across all age groups mirrors the lack of genotype 4 in the general Moroccan population. Authors acknowledge the insufficient number of subjects in some age intervals, and further studies with more patients need to be done in order to accurately assess the uniformity of genotypes in heroin addicts of all ages.

We next sought to determine if our IDU cohort had a higher viremia compared between the different genotypes. ANOVA analysis of genotype and average viremia (IU/mL)) was significant (F = 2.98, p = 0.0412, *n* = 49), indicating that individuals infected with genotype 1a had a higher viral load titer as compared to genotypes 1b, 3a, and 4a respectively (Figure 2). Our results conflict with a previous study that did not observe a higher viremia associated with genotype 1a across all age groups (Schijman et al.) [40].

Further complicating the picture is the co-infection with Hepatitis B virus (HBV) and Human Immunodeficiency virus (HIV-1), which ultimately alters the natural course of the disease. It is now well-established that a substantially higher viral load is present in HIV-1 co-infected individuals as compared to HCV mono-infected patients (Matthews-Greer et al.; Cribier et al.) [41,42]. HBV/HCV dual-infection results in cross-suppression of viral replication, as well as rapid progression of liver disease and cirrhosis, while HIV-1/HBV co-infected individuals often exhibit immune suppression and higher viral RNA loads (Sulkowsky; Filippini) [43,44], mirroring the situation in HIV-1/HCV co-infected patients. A possible next step is to address the proportion of HCV-positive Moroccan IDUs co-infected with either HIV-1 or HBV.

It is widely recognized in the medical community that different genotypes respond varyingly to antiviral therapy and that no standard of care (SOC) treatment exists for all 6 genotypes (Petta & Craxi; Grassi & Aghemo; Esmat et al.) [45-47], with the ‘gold standard’ treatment currently being pegylated interferon-α (pegINFα) in combination with ribavirin (RBV) for 24 to 48 weeks. The addition of protease inhibitors Boceprevir and Teleprevir implemented in triple therapy significantly increases response rates and sustained virology response (SVR), however this comes at the price of additional side effects and the risk of therapy discontinuation (Chopra et al.) [48]. Studies are at odds in terms of the efficacy of antiviral treatment based on genotype: genotype 1a has been shown to be negatively correlated to SVR (Petta & Craxi) [45], while another study reported a better response rate to dual antiviral therapy in mono-infected patients using Boceprevir and Telaprevir (Pelicelli et al.) [49]. Generally speaking, genotype 1b associated with a more aggressive disease course, and patients are more likely to exhibit liver cirrhosis and decompensated liver disease (Zein) [50]. Better response rates are obtained in genotype 2 and genotype 3 patients (about 80%), while genotype 1-infected patients remain the hardest to treat overall, reaching an SVR of only about 40% (Jazwinski & Muir) [51]. With respect to genotype 3 infected individuals, the above mentioned SOC treatment was tailored to 24 weeks and was shown to be effective in terms of SVR, and further shortened to 12-14 weeks for those patients exhibiting rapid virologic response (RVR) (Andriulli et al.; Zeuzem et al.) [52,53]. In light of these findings, the treatment implications for the Moroccan IDU cohort are somewhat positive, seeing that the majority are infected with either genotypes 1a or 3a. However, the medical community must first tackle the issue of treatment refusal before effective therapy can be implemented, as IDUs have been known to decline medical help (Mehta et al.) [54]. For it to be successfully resolved, the issue must be approached on multiple fronts: reaching out to drug addicts as they are often marginalized from mainstream society; ensuring that IDUs are aware of various treatment options at their disposal; undertaking a cost-benefit analysis for each patient in order to choose the best course of action and a tailored therapy.

## Conclusion

This is the first national study on the genotype profile of Moroccan IDUs living in the northern region of Morocco, and highlights the fact that they are a distinct cohort and display different demographic, serologic, and genotypic profiles. Consequently, IDUs should be considered apart from the HCV-infected pool derived from the general population and other high risk groups, There is brewing hope for worldwide HCV elimination, with the strategy using a ‘cocktail’ consisting of transmission prevention, genotype-tailored treatments, routine blood-product screening, and vaccine development (Hegan & Schinazi) [55]. Moroccan health authorities must implement public health campaigns to address this issue of high seroprevalence in IDUs, as well as using prevention as their primary tactic for disease control. Finally, combatting the HCV epidemic entails a joint effort between health practitioners and local governments, employing a continuous epidemiological surveillance.

## Materials and methods

### Patient recruitment and selection criteria

This research is a retrospective study and a collaborative effort between the Hasnouna Center for Addictology in Tangier and the Pasteur Institute of Morocco in Casablanca between the years 2011 to 2013. All subjects included in the study fall into two distinct categories: registered IDU patients at the Hasnouna Center who are currently undergoing detoxification and/or therapy for their drug (heroin) habit and are on methadone treatment, or chronic HBV carriers that are being treated with antiviral therapy at the Pasteur Institute of Morocco. Drug addict patients all had an ‘active’ drug addiction and were presumed to be representative of the general intravenous drug user population in Morocco, thereby serving as a source of data extrapolation. Prior to enrolment of subjects, an internal ethics committee at the Pasteur Institute reviewed the study and approved it as a human subject study that did not violate or harm subjects in any way. IDU patients were first recruited and evaluated by two attending physicians at the Hasnouna Center, during which the objectives of the study were explained to each patient by the attending physicians, and no undue coercion was exercised if the patient refused to participate. There were no minors recruited and each patient signed an informed consent form demonstrating his or her willingness to participate in the study. After which, a follow-up visit was scheduled during which: blood was drawn, data for the various variables was collected, and treatment was initiated (methadone). Patient blood samples which tested positive for anti-HCV antibodies were frozen in sterile bags and sent to the Molecular Biology Laboratory at the Pasteur Institute of Morocco for viral RNA detection, viral load quantification, and genotyping. Chronically-infected HCV patients (non-IDUs) who were used as the comparator population are currently having their regular blood tests done at the Pasteur Institute of Morocco. All subjects have signed an informed consent form in which they allowed their personal data and lab results to be used for any research purposes.

### Samples

Blood sera from 211 IDU patients were collected at the Hasnouna Addiction Center in Tangier from 2011 to 2013, aliquoted (EDTA) and stored at -20 C until further use. Blood sera (*n* = 151) from chronically infected patients were collected at the Pasteur Institute of Morocco in 2013, aliquoted (EDTA), and stored at -20 C until further use.

### RNA detection and genotyping

Viral RNA extraction was done using COBAS Ampliprep™; viral load quantification was performed using real-time RT-PCR 48 HCV test (Cobas Ampliprep/Cobas TaqMan™ (RT-PCR), Roche Diagnostics, Pleasanton, USA) with a limit of detection of 15 IU/mL, all samples above this limit were considered HCV positive; Viral RNA was reverse transcribed and amplified with Versant HCV Amplification 2.0 kit (LiPA) (using 26 μL of amplification mix, 4 μL of enzyme mix, 20 μL of viral RNA) and using GeneAmp PCR System 9700 (Applied Biosystems, Life Technologies, USA). Viral samples were genotyped using Versant HCV Genotype Assay version 2.0 (Siemens). All tests were performed according to the manufacturer’s instructions.

### Statistical analyses

All data was analyzed using statistical software R (version 3.0.1): χ^2^, ANOVA, independent sample T-test, and Pearson’s Correlation. For all tests, all p values less than 0.05 were considered statistically significant.

## Abbreviations

HBV: Hepatitis B virus; HCC: Hepatocellular carcinoma; HCV: Hepatitis C virus; HIV-1: Human immunodeficiency virus; IDU: Intravenous drug user; IDUs: Intravenous drug users; MOHP: Minister of health and population (Egypt); PegINFα: Pegylated interferon alpha; RBV: Ribavirin; RVR: Rapid virologic response; SOC: Standard of care; SVR: Sustained virologic response.

## Competing interests

The authors declare that they have no competing interests.

## Authors’ contributions

RDT made substantial contributions to genotyping, literature review, data analysis and statistics, and manuscript writing. FZS participated in sample collection and data compilation. FL, WB, AF, AE participated in genotyping, viral RNA extracting and detection. MS was responsible for the overall supervision of the study. AMB was responsible for study oversight and coordination. AB was responsible for study conception and design, supervision of all laboratory work, review of manuscript and proof-reading. All authors read and approved the final manuscript.
